# RNA-seq analysis of single bovine blastocysts

**DOI:** 10.1186/1471-2164-14-350

**Published:** 2013-05-25

**Authors:** James L Chitwood, Gonzalo Rincon, German G Kaiser, Juan F Medrano, Pablo J Ross

**Affiliations:** 1 Department of Animal Science, University of California, Davis, 1 Shields Avenue, Davis, CA, USA; 2 Laboratorio de Biotechnología de la Reproducción, INTA, Balcarce, Argentina

**Keywords:** RNA-seq, Bovine, Embryo, Blastocyst, SNP, ASE

## Abstract

**Background:**

Use of RNA-Seq presents unique benefits in terms of gene expression analysis because of its wide dynamic range and ability to identify functional sequence variants. This technology provides the opportunity to assay the developing embryo, but the paucity of biological material available from individual embryos has made this a challenging prospect.

**Results:**

We report here the first application of RNA-Seq for the analysis of individual blastocyst gene expression, SNP detection, and characterization of allele specific expression (ASE). RNA was extracted from single bovine blastocysts (n = 5), amplified, and analyzed using high-throughput sequencing. Approximately 38 million sequencing reads were generated per embryo and 9,489 known bovine genes were found to be expressed, with a high correlation of expression levels between samples (r > 0.97). Transcriptomic data was analyzed to identify SNP in expressed genes, and individual SNP were examined to characterize allele specific expression. Expressed biallelic SNP variants with allelic imbalances were observed in 473 SNP, where one allele represented between 65-95% of a variant’s transcripts.

**Conclusions:**

This study represents the first application of RNA-seq technology in single bovine embryos allowing a representation of the embryonic transcriptome and the analysis of transcript sequence variation to describe specific allele expression.

## Background

Transcriptome sequencing describes global trends in gene expression while also detailing alterations to biological pathways at the gene-specific level [1-3]. High-throughput sequencing of RNA (RNA-seq) quantifies transcripts expressed from known genes with an enormous dynamic range and discovers transcriptional units that are biologically novel yet previously unannotated, or not fully characterized in available databases or gene expression arrays [4,5]. The localization of transcript sequence to different areas of a gene (exon, intron, UTR’s) at base-pair resolution can detect instances of alternative splicing and transcript isoforms while sequencing of multiple RNA species allows for detection of many genetic regulatory elements of biological significance [6-8]. These advantages make RNA-seq a suitable tool for examining the biology of preimplantation embryos. While single cell transcriptome sequencing is becoming more common, sequencing of individual embryos is not typically performed due to a scarcity of biological material necessary for sequencing library preparation. This limitation has previously been overcome by pooling of embryos, which precludes important aspects of genome biology from being examined; namely analysis of an individual sample’s genetic variation and identification of allele-specific expression (ASE). The ability to ascertain expression of known and novel SNP and to detect imbalanced allelic expression, in addition to discrete quantification of genome-wide transcript abundance, give RNA-seq of individual embryos enormous utility in studying early developmental processes.

Detection and categorization of SNP within production animal systems has been performed extensively [9]. Use of these variants as markers and predictors of performance in a large variety of traits (i.e. fertility, milk production, calving ease, etc.) is common [10-12]. In the context of early development, classifying SNP between samples of varying viability, sex, or breed allows for discovery of novel markers of fertility and characterization of critical regulatory mechanisms of embryonic development, such as epigenetic reprogramming or embryonic genome activation. Use of transcript sequence for variant detection has been performed with various assays [13], but also recently in cattle using RNA sequencing data [14]. While identification of sequence variation identity at nucleotide resolution and position is valuable, determination of allelic imbalances (AI), defined as a shift from a 50:50 expressed ratio, require transcript sequencing of single samples. Expression imbalances have been associated with variation in performance traits and disease processes [13,15], and could be relevant to embryonic development [16]. AI provides another means of genetic regulation and is a characteristic of imprinted genes, although detection of these with transcript sequencing remains controversial [17,18].

To examine the feasibility of transcript sequencing of single embryos we performed RNA-seq of bovine IVF-derived blastocysts. Particular importance was placed on the analysis options available for this type of data and consideration was given to sequence read processing, bovine genome assemblies available for alignment, and mapping strategy. Transcriptome profiling of replicates was found to be highly reproducible and genes and pathways associated with embryonic samples were highly expressed or enriched. Variant analysis was also performed with in silico validation of detected SNP to define criteria for discerning true variation from sequencing artifacts. Similarly, statistical tests and skew thresholds were defined for classification of AI. The ability to locate genetic variation on a global scale and also quantify the expression of allelic variants demonstrates the unique advantages afforded by sequencing of individual pre-implantation embryos.

## Results and discussion

### Sequencing library preparation

**Figure 1:**
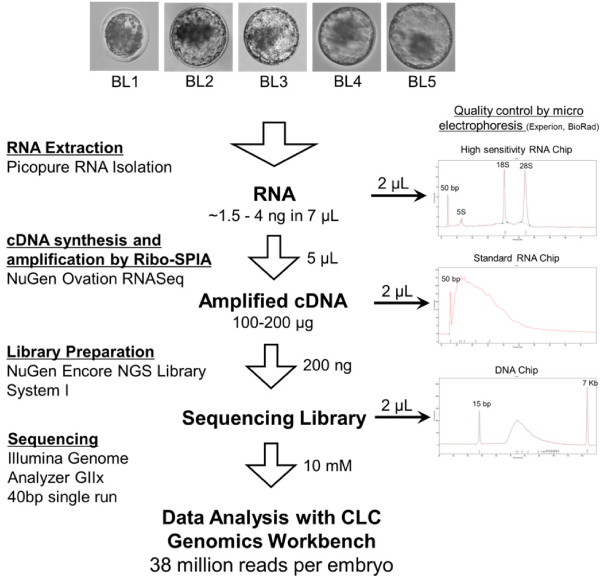
**Workflow of bovine blastocyst sequencing preparation.** RNA from Day 7 blastocysts was extracted, converted to cDNA and amplified before being processed with the Nugen Encore Library System. Electropherograms on right depict quality controls performed at each of the steps.

The initial obstacle to performing RNA-seq from a single blastocyst is obtaining sufficient amounts of high quality RNA for use in sequencing library preparation. To accomplish this we used a standard column-based method of total RNA isolation, including a DNAse treatment, and eluted the RNA into a small volume to achieve a concentration suitable for amplification and library preparation. We obtained between 1.3 and 2.1 ng of total RNA per embryo (Figure 1). These amounts correspond closely to other reports using an identical methodology [19] and the same RNA extraction kit [20,21] used presently. Examination of the 18S and 28S rRNA fractions by micro-electrophoresis showed profiles similar to those previously observed in total RNA derived from bovine blastocysts [19,20] and possessing high RQI quality scores (>9). The amount of RNA harvested from a single embryo is not sufficient for preparation of sequencing libraries using standard methods and necessitates amplification following cDNA synthesis. Methods for RNA amplification have been developed including PCR, in vitro transcription (IVT), and Ribo-SPIA (single primer isothermal amplification)-based amplification methods. We chose the latter approach given that it is recommended for the levels of RNA input we obtained from blastocysts and requires only one round of amplification to produce sufficient amounts of material for downstream library preparation.The Ribo-SPIA amplification method is based on an isothermic reaction where transcription initiation sites are primed with random and oligo-dT primers and synthesis of single-stranded cDNA occurs via degradation of RNA in a DNA:RNA primer hybrid to create a template permitting multiple instances of transcription [22,23]. This method has been extensively validated in microarray [24], large-scale RT-PCR [25], and recently RNA-seq [26] studies.

After RNA amplification starting from ~1 ng of total RNA, we obtained on average 6 μg of cDNA. Amplification produced cDNA fragments with an average size range of 200 to 300 bp, therefore physical or enzymatic fragmentation and size exclusion by gel electrophoresis was not necessary. No difference in transcript coverage was observed when libraries were prepared from sheared or non-sheared cDNA from the Ovation system in other studies [26]. The cDNA output from the Ovation Amplification kit was then used for sequencing library preparation, at which point sequencing adapters are ligated to cDNA fragments. Electropherograms obtained from amplified cDNA and sequencing libraries indicated that high quality libraries were obtained (Figure 1). Previous attempts to generate RNA-seq libraries from single bovine embryos were unsuccessful [27], this probably resulted from the lower output of amplified material generated by the IVT-based approach, even when two rounds of amplification were performed. Conversely, the SPIA approach has been shown to produce higher outputs while maintaining a linear amplification pattern [28].

Normally, the high proportion of ribosomal RNA (rRNA) compared to mRNA represents a problem for RNA-seq studies, requiring steps aimed at eliminating the rRNA fraction before library construction and sequencing. However, human and mouse samples prepared using the Ovation amplification system contain low percentages of rRNA fragments (NuGen, personal communication; [26]). Alignment of unmapped reads to a non-coding RNA database (RFAM) resulted in a 0.2% alignment rate, confirming that the bovine embryo libraries prepared using the Ovation V1 system did not contain large proportions of rRNA fractions. While the exact mechanism by which rRNA are not amplified is currently unknown, it is speculated that the secondary structure of rRNA is likely responsible for inefficient primer binding resulting in low cDNA conversion efficiency and minimization of rRNA amplification.

It is also important to note that this amplification methodology excludes small RNA populations below 50 bp in size, such as microRNAs, meaning that a variety of small non-coding RNAs with potential significance to the blastocyst are not included in the sequencing data. As a confirmation of this, miRNA transcripts were not detected in any of our samples. Overall, RNA extraction from samples with typically restrictive amounts of RNA using a column-based extraction and amplification with Ribo-SPIA allowed for the preparation of high quality sequencing libraries from single bovine blastocysts.

### Sequence read processing

**Figure 2:**
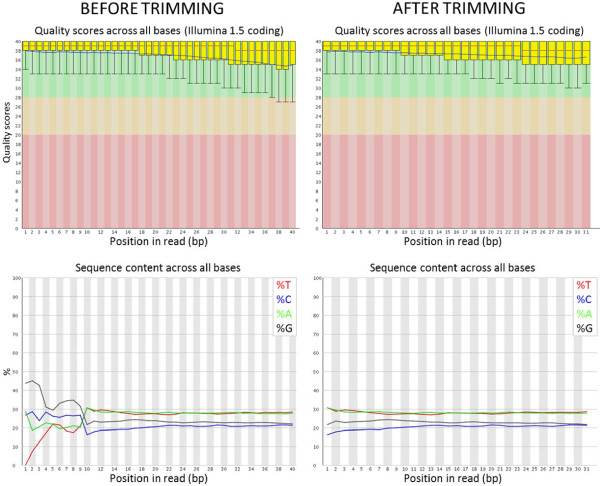
**Galaxy QC outputs for a representative sample.** Read quality across the sequence was acceptable on average but G/C content was abnormally high at the first 9 bp of the 5′ end (lower left box). Trimming of sequences (two boxes on right) removed this bias.

On average, 38 ± 1.1 million single reads of 40 bp were generated per embryo with an Illumina GAIIx sequencer. Initial mapping of unprocessed reads to the bovine genome (BTAU4.0) allowing for two mismatches resulted in only 69.1% of total reads mapping. Allowing for the possibility of low quality reads at the ends of sequences, we evaluated read quality using Galaxy (Figure 2). No evidence of low quality reads based on Illumina’s scoring was observed spanning the 40 bp of sequence (Figure 2). However, base sequence content across the reads indicated that the 5′ ends had a higher proportion of G and C nucleotides that deviated from the rest of the read. This abnormal pattern spanned the first 9 bases sequenced. A potential source of this problem could be that the Ovation RNA amplification system uses 9mers to perform the RT and are likely to have high GC content. Also, since amplified reads are not shared, and the sequencing primer is ligated to one end of a read, it would be expected by chance that half of the sequences generated begin sequencing at the 9mer annealing site. Moreover, it is possible that 9mer primer binding is not 100% efficient and thus could incorporate mistakes to the sequence. Thus, we hypothesized that removing the first 9 bp of the reads would improve mapping by removing bad sequence.

We tested if removing nucleotides at both or either end, and especially nine bp from the 5′ end, would enhance the proportion of reads mapping to the genome (Table 1). Trimming 2 nucleotides at each end of the reads significantly improved the % of read alignment from 70 to 82%. This was not the result of just removing 4 bp from the reads, since trimming 4 bp at the 3′ end results in a lower read alignment of 71%. Similarly, removing 9 bp from the 3′ end resulted in a low alignment percent (73.27%), although this was higher than untrimmed read mapping probably as a result of the reduced mapping specificity of shortened reads. Trimming 4 bp and 9 bp from 5′ end resulted in a larger improvement in alignment percentages (84 and 89%, respectively), which is likely the result of removing low quality information from the reads. Based on these results, we performed all other analyses with reads trimmed 9 bp at the 5′ end.

**Table 1:** Effect of end trimming on read alignment to genome

**5′ Trim (bp)**	**3′ Trim (bp)**	**% reads mapped to bovine genome (BTAU4.0)**					
		**BL 1**	**BL 2**	**BL 3**	**BL 4**	**BL 5**	**Mean ± SEM**
**0**	**0**	67.9	70.1	70.0	67.7	70.0	69.1 ± 0.5
**2**	**2**	80.7	82.8	82.5	81.7	82.5	82.0 ± 0.4
**0**	**4**	69.7	72.6	71.9	69.7	72.0	71.2 ± 0.6
**0**	**9**	72.0	74.6	73.9	71.8	74.0	73.3 ± 0.6
**4**	**0**	83.4	84.2	85.0	84.3	84.9	84.4 ± 0.3
**9**	**0**	88.0	89.4	89.6	88.7	89.3	89.0 ± 0.3

Processing of reads prior to alignment by removal of this priming sequence not only significantly improved the fidelity of read alignment, but also the accuracy of SNP analyses (data not shown). Since the time of these experiments, Nugen has incorporated a cDNA fragmentation step after amplification, minimizing the chance that a fragment will be sequenced at the primer binding site and thus this trimming may not be necessary in future libraries. However, our experience indicates that quality control of reads is important and removing affected nucleotide positions, although reducing the amount of total sequence, can greatly enhance the accuracy of the results.

### Mapping of reads to different genome annotations

Multiple assemblies and annotations of the bovine genome exist from different sources (e.g. Ensembl, NCBI, UCSC, UMD). Since these annotations contain different numbers of genes and transcripts we compared general mapping statistics after mapping trimmed reads of each embryo to the annotated assemblies using the RNA-seq algorithm of CLC Genomics allowing for 2 mismatches (Table 2). The differences in total reads aligned varied by as much as 6 million (or 16%) when comparing NCBI-Btau 4.2 and Ensembl-UMD3.1 (61 vs. 77% total mapped reads, respectively). The difference in the proportion of uniquely aligned reads was even greater between NCBI-Btau 4.2 and Ensembl-UMD3.1 (48 vs. 68%, respectively). These large differences were observed even though both annotations contained a similar number of genes. Also, differences were observed when comparing different annotations of the same genome assembly (NCBI-UMD3.1 vs. Ensembl-UMD3.1). The Ensembl annotation of UMD3.1 resulted in higher proportion of reads mapping even though this annotation contained fewer genes than the NCBI annotation. These mapping read statistics indicate Ensembl-UMD3.1 is a more comprehensive genome annotation, not only in terms of identifying actual genes, but through its increased ability to uniquely localize reads. It is possible that these annotations contain different proportions of embryonically expressed genes and thus alternative annotations may be better for different tissues. Increased alignment to annotated genes improves the robustness of RNA-seq results, thus we selected the Ensembl-UMD3.1 annotation for further analysis.

**Table 2:** Number and proportion of reads mapped to different genome annotations and different genome builds

	**Annotation source and genome build**			
	**NCBI Btau 4.2**	**NCBI UMD 3.1**	**Ensembl Btau 4.0**	**Ensembl UMD 3.1**
Number of genes in annotation	24,359	25,577	25,670	24,616
Number of transcripts in annotation	19,757	20,681	26,977	22,118
Mean number of reads processed per embryo (Million reads ± SEM)	37.6 ± 1.1	37.6 ± 1.1	37.6 ± 1.1	37.6 ± 1.1
Total number of reads mapped to annotated genes (Million reads ± SEM)	22.9 ± 0.6	24.6 ± 0.6	27.0 ± 0.7	28.9 ± 0.8
% total reads mapped	60.9%	65.6%	71.9%	76.9%
Number of uniquely mapped reads (Million reads ± SEM)	18.0 ± 0.5	18.2 ± 0.6	23.8 ± 0.7	25.5 ± 0.7
% reads uniquely mapped	48.1%	48.4%	63.2%	67.7%

### RNA-seq mapping statistics

**Figure 3:**
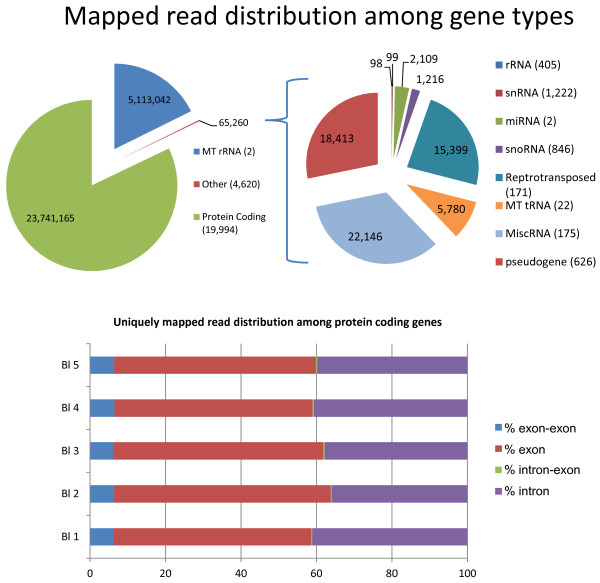
**Distribution of mapped reads to different transcript types and gene regions.** Top graph indicates the proportion of transcripts belonging to different RNA species. Numbers within pie chart indicate number of reads while numbers in parenthesis indicate number of transcripts. Bottom graph shows the read distribution within protein coding genes.

Of the total reads, an average of 76.9% (approximately 29 million reads per sample) mapped to annotated portions of the Ensembl-UMD3.1 genome. Among these, 81.9% of the reads mapped to protein coding genes, 17.6% to mitochondrial rRNA and the other 0.2% was distributed between miscellaneous RNAs, pseudogenes, retrotransposed elements, and mitochondrial tRNAs (Figure 3). The presence of high levels of mitochondrial rRNA and tRNA was in agreement with the high level of expression of mitochondrial protein coding genes. Out of the 13 protein coding genes present in the mitochondrial DNA, 9 were the most highly expressed genes among all protein coding genes and the other 4 ranked at positions 15, 16, 19 and 57, among a total of 19,994 protein coding genes. This indicates that a large number of blastocysts transcripts are coded by the mitochondrial genome. Also, it is not clear why mitochondrial, but not nuclear, rRNA is detected by the methodology used. It could be that the three dimensional structure of mitochondrial rRNA does not prevent random primer binding and is amplified. On the other hand, the amount of mitochondrial rRNA detected does not interfere with analysis of protein coding RNAs, since as indicated above, most reads mapped to protein coding genes.

Among reads mapping uniquely to protein coding genes (~20 million reads per embryo), 60% were located to exons, including 6% to exon-exon boundaries. 39% were located to introns and 0.4% to exon-intron boundaries (Figure 3). The relatively high proportion of reads assigned to introns is not uncommon when the sequencing library preparation includes random priming of the mRNA [26]. This is not often seen when the RT reaction is performed using oligo-dT primers and only amplifying polyadenylated transcripts. Whether these intron sequences belong to un-processed transcripts or un-annotated exons is not clear and deserves further investigation. Towards the latter possibility, CLC Genomics identifies and quantifies the presence of putative exons and we identified an average of 9,684 putative exons per sample. Analysis of the location and identity of these potentially novel coding sequences goes beyond the scope of this manuscript but warrants additional attention.

**Figure 4:**
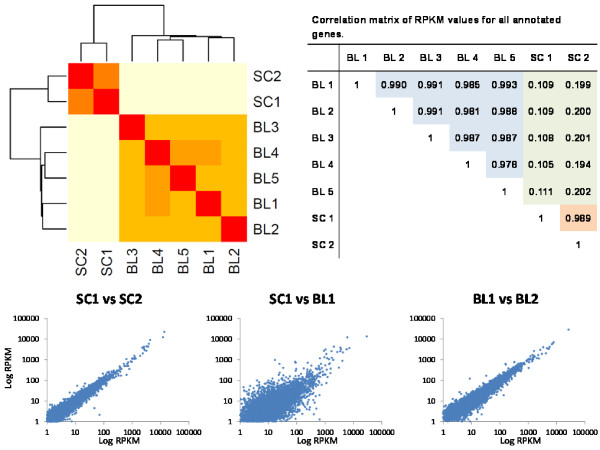
**The heat map shows the segregation of samples by gene expression.** Blastocyst (BL1 to BL5) and milk somatic cell (SC1 and SC2) transcriptomes are distinguished clearly from one another while grouping highly within their own sample types. Average correlation of RPKM for all annotated genes reflected this within BL (r > 0.97) and between sample groups (r < .21) and plotting of log-transformed RPKM values showed a much less linear relationship between milk somatic cells and blastocyst samples.

Of the 19,994 protein coding genes in UMD3.1, 9,155 ± 107 (46%) were expressed per embryo with at least 0.4 RPKM, a value slightly more conservative than the 0.3 RPKM threshold suggested to represent above background expression levels [29]. The maximum RPKM value was 37,015, indicating a dynamic range of expression spanning 6 orders of magnitude. The correlation of RPKM values considering all genes evaluated was high among individual blastocysts (r > 0.97), indicating the consistency of this methodology (Figure 4). Milk somatic cell samples analyzed with a similar approach (same RNA amplification, library preparation and data analysis) had a high correlation among the replicates (r = 0.99). On the other hand, the correlation between embryos and somatic cells was extremely low (r < 0.21). These results indicate a high correlation between biological replicates but not between samples of different origin, suggesting that discrimination between samples is possible based on whole transcriptome analysis.

The number of genes detected in our study could be considered low when contrasted to those from other bovine blastocysts studies (17,634 [27] and 22,170 [30]). One important consideration is that our analysis results in number of genes expressed while other publications usually report number of transcripts. Given that multiple transcripts can results from the single gene, the comparison of number of expressed genes to number of expressed transcripts should be made cautiously. Also, it is important to note that the threshold used for considering a gene detected can have large implications in the number of detected genes. We selected an RPKM > 0.4 as a conservative approach to the recommended 0.3 [29]. Reducing the RPKM threshold results in detection of 16,150 genes, which is in line with what is reported elsewhere [27,30].

We compared our RNA-seq results from single embryos to those generated by Huang and Khatib using pools of 20 normal bovine blastocysts (GEO accession GSE25082). Raw data was downloaded and processed according to our bioinformatics pipeline. At RPKM > 0.4 a total of 11,501 genes were detected in the pooled blastocysts dataset, a similar number of genes (11,039) was also detected in our dataset, when considering it expressed in at least one embryo. Among these, 7,247 genes were commonly expressed in all datasets. When we considered genes consistently expressed in all 5 unique embryo samples, 7,526 transcripts were detected, out of which 7,247 (96%) were also present in the Huang and Khatib dataset. Also, principal component analysis (PCA) and hierarchical clustering of all RPKM values indicated that pooled and single blastocysts clustered together and apart from milk somatic cell samples (Additional file 1: Figure S1). These results indicate a highly similar representation of blastocyst transcripts between the studies; in spite of different embryo production, RNA extraction, amplification, and library preparation methodologies. Furthermore, the similarity in results highlights the robustness of RNA-seq analysis.

The average median coverage of annotated exons with aligned reads was 5.43X. This coverage corresponds closely to a depth necessary to detect 73% of moderately abundant transcripts (15–30 RPKM) and identify gene isoforms and novel splice junctions [8]. Gene isoforms derived from alternative splicing are a significant contributor to extra-genomic variation [31] and exon-exon junctions have been used to identify instances of alternative splicing [32,33]. Exon-exon junctions contributed on average 11.4% of the total aligned exon reads and intron-exon reads, potentially representing pre-mRNAs, constituted a very low proportion (0.4%) of reads. This subset of possible regulatory isoforms could lead to discovery of developmentally-related forms of gene regulation unrecognized in non-embryonic tissues and examination of these isoforms between embryonic developmental stages is needed to determine their significance.

### Functional annotation of blastocyst expressed genes

We performed functional annotation of genes based on level of expression. Genes expressed in all 5 blastocysts were sorted by average RPKM into 1) High; 2) Medium High; 3) Medium; 4) Medium Low; and 5) Low expression level groups. Functional categories enriched in each group compared to the genome (P < 0.01) are shown in Table 3. Enrichment of constitutive cellular elements such as the cytoskeleton, ribosomes and mitochondria was prevalent in higher expression groups, indicating that the blastocysts are rapidly synthesizing proteins to sustain a high rate of cell division and growth. Also, all proteins required for oxidative phosphorylation were in the high expression level clusters, suggesting this mechanism of energy production is very active in blastocysts. High and Medium High brackets mostly related to cell structure and basic cellular biology functions, respectively. In Medium expressing groups, transcription and related processes (DNA binding, zinc fingers) were prominent, while in the lower expression brackets, more nuanced aspects of transcription/translation regulation were found to be enriched (tRNA and zinc finger proteins). Processes of the lower expression brackets were more varied and functionally specific as evidenced by the presence of specific DNA repair mechanisms and regulators of cell death, reflecting the greater variety of cellular functions represented outside of the constitutive structural functions found in the Medium and High expression level groups.

**Table 3:** Summary of GO enriched clusters in groups of genes sorted by expression level in blastocysts

**Cluster #**	**Expression level**				
	**High**	**Medium high**	**Medium**	**Medium low**	**Low**
1	Ribosome	Lumen	Lumen	Lumen	DNA repair/Stress response
2	Oxidative Phosphorylation	Cytoskeleton	Chromosome structure	Zinc finger PHD	Zinc/ion binding
3	Ubiquitination	Protein catabolism	Protein complexes	Transcriptional regulation	Protein catabolism
4	Spliceosome	Chromosome structure	Mitochondrial subunits	Mitochondrial matrix	Protein transport
5	RNA binding	Nucleic acid binding	Nucleic acid binding/Phospho-rylation	Ankyrin repeats	tRNA aminoacylation
6	Electron transport	Ubiquitination	Endoplasmic reticulum	Protein/nucleotide interactions	Zinc finger CH2
7	Protein localization	Protein complex synthesis	Cell cycle	Microtubules	Cell death
8	Protein biosynthesis	Cell cycle	Cellular membranes	Mitochondrial membranes	DNA repair/Polymerase
9	Ribosomal subunits	Mitochondrial components	Ankyrin repeats	Phosphorylation	Suppression of differentiation
10	Protein folding	RNA splicing	tRNA	Zinc finger CH2	Nucleic acid binding

To identify overexpressed genes in embryos the transcriptomes of blastocysts and milk somatic cells (data from [34]) were compared. The choice of milk somatic cell as a reference for comparison was made based on the availability of the data and its expected dissimilarity in global gene expression. As expected, we identified large differences between these samples (Figure 4) with a total of 4,952 genes differentially expressed at an adjusted P value < 0.001. Among these genes, 621 were overexpressed in the blastocyst samples by at least a 10 fold-change difference. This group of blastocyst-overexpressed genes was subjected to gene ontology classification and functional analysis using DAVID. The top overrepresented biological processes corresponded to embryonic development, including blastocyst development (Table 4). The blastocyst stage embryo is composed of inner cell mass (ICM) and trophectoderm (TE) cell lineages. The ICM is a pluripotent cell lineage that gives rise to all the tissues in the embryo proper, and from which embryonic stem cells are derived. As such, stem cell maintenance was among the top overrepresented categories. The TE differentiates into placental tissues, and placental development was another one of the top overrepresented biological processes. Interplay between the ICM and TE ensures that these tissues are correctly specified.

**Table 4:** GO biological process representative of top ten overrepresented functional annotation clusters among genes overexpressed in embryos versus somatic cells at P < 0.001 and 10 FC

**Biological process**	**Gene count**	**P value**	**Genes**
Chordate embryonic development	27	4.6E^-6^	*HNF1B, CDX2, PTK7, CITED1, ZIC2, GATA2, DAB2, POU5F1, RSPO3, TDGF1, KRT8, HOXC5, AXIN2, GINS1, GSC, TBX3, ESRRB, NASP, GJB5, SLC34A2, CCNB1, HOXB4, GCM1, SALL4, PDGFRA, HOXB9, MYH10*
Blastocyst development	6	0.007	*GINS1, HNF1B, CDX2, ESRRB, POU5F1, NASP*
Pattern specification process	16	0.002	*NANOG, HNF1B, GSC, CDX2, TBX3, OTX2, ZIC3, TCF7L1, HOXB4, LHX2, FOXG1, HOXC5, TDGF1, HOXB9, HHIP, AXIN2*
Negative regulation of gene expression	21	0.001	*DNMT3A, NANOG, GSC, CDX2, TBX3, RCOR2, SOX2, MLXIPL, MAEL, CENPF, TNP1, LIN28A, PKIA, LIN28B, TCF7L1, HOXB4, SALL4, POU5F1, SOX15, DNMT3B, TDRD1*
Placenta development	9	8.5E^-4^	*GATA2, CDX2, GCM1, ESRRB, RSPO3, KRT8, GJB3, GJB5, CITED1*
Cellular component morphogenesis	23	2.1E^-5^	*CCDC99, ACTC1, NDN, ESRRB, PDPN, MYBPC3, KIF5C, PTK7, MAEL, SLITRK2, LAMA1, DAB2, BDNF, LAMB2, PRDM14, FAT1, LHX2, FOXG1, KRT8, SLITRK5, NEFL, MYH10, GFRA3*
Stem cell maintenance	5	0.001	*NANOG, CDX2, ESRRB, POU5F1, SOX2*
Cell adhesion	24	0.005	*TYRO3, CADM3, MPZL2, ATP1B2, CLSTN3, PDPN, CLDN6, MYBPC3, FERMT2, PTK7, BCAM, AMIGO2, LAMA1, LAMB2, DSG2, SORBS1, DSG3, PKP2, FAT1, FREM1, PECAM1, COL12A1, CNTNAP1, ESAM*
Establishment of organelle localization	5	0.007	*CCDC99, NPM1, CENPF, CDCA5, MYH10*
Regulation of glucose metabolic process	4	0.003	*HNF4A, SORBS1, MLXIPL, GNMT*

Specific transcription factors need to be expressed to differentiate embryonic cells into these specific tissues, and transcription factor activity was the most overrepresented molecular function. Among them, *OCT4*, *NANOG* and *SOX2* are well known transcription factors associated with pluripotency and were all expressed in embryos, but not in milk somatic cells. *SALL4*, a gene known to regulate OCT4 expression in mice was detected. *CDX2* a transcription factor required for TE development was highly expressed in embryos. Also, *GATA2*, a known regulator of trophoblast lineage transcription was present in all five samples, as well its upstream regulator *TEAD4*. *IFNtau*, the factor responsible for pregnancy recognition in cows, was also present. *GRB2* is a known suppressor of *NANOG* in the primitive endoderm (PE) of the inner cell mass (ICM), and homozygous mutants of *GRB2* are believed to arrest shortly after implantation [35]. *GRB2* and *GATA6* were expressed in all samples, which suggest that primitive endoderm differentiation may already be active in day 7 embryos. *GATA3*, *TEAD4*, and *GRB2* suppress expression of *NANOG* and *OCT4* and are thought to diminish pluripotent gene expression to create and maintain extra embryonic lineages [36,37]. Establishment of new embryonic cell fates requires epigenetic changes, and de novo DNA methylation genes (*DNMT1A* and *DNMT1B*) were highly expressed in embryos. Also, post-transcriptional regulatory mechanisms are important for regulating cell fate changes, and *LIN28*, a factor required for embryonic stem cell differentiation through regulation of miRNA activity, was highly expressed in blastocysts.

To create the blastocoel cavity the trophectoderm cells form a polarized epithelium and transport ions across them, generating osmotic pressure that moves water into the blastocoel. Cell-cell junction related genes were highly expressed, including genes associated with GAP junctions and desmosomes. Also, ATPase activity mostly associated with ion pumps such as the Na+/K + (*ATP1B2*, *ATP1B3*) and H + (*ATP6V0A4*, *ATP6V1G3*) ATPases were among the top overrepresented molecular functions in blastocyst genes (Table 5).

**Table 5:** GO molecular functions representative of top five overrepresented functional annotation clusters among genes overexpressed in embryos versus somatic cells at P < 0.001 and 10 FC

**Molecular process**	**Gene count**	**P value**	**Genes**
Transcription factor activity	33	0.002	*HNF1B, CDX2, SOX2, DMRTA2, TCF7L1, CITED1, MSX2, GATA2, POU5F1, LHX2, HOXC5, SPIC, LHX8, PITX1, KLF5, NANOG, GSC, TBX3, RCOR2, ESRRB, OTX2, ESRRG, TEAD3, FOXR1, MYCN, HOXB4, GCM1, HNF4A, LASS3, FOXG1, DLX4, HEYL, HOXB9*
ATPase activity, coupled	14	0.003	*RECQL4, ATP1B3, ATP1B2, DDX4, DDX28, DDX3Y, ABCC4, ATP6V1G3, ABCC2, ATP6V0A4, KATNAL2, ABCC5, ATP7B, MYH10*
Transmembrane receptor protein tyrosine kinase activity	7	0.002	*IGSF10, TYRO3, FGFR4, FGFR3, PTK7, PDGFRA, KIT*
Folic acid binding	3	0.028	*FOLR1, SLC19A3, GNMT*
Helicase activity	8	0.037	*RECQL4, DDX28, PIF1, TDRD9, DDX3Y, MCM4, RAD54L, DDX4*

### Sex-specific gene expression profiles

Differences in gene expression have been detected between blastocysts of different gender. In an attempt to determine the sex of the embryos we analyzed expression of candidate sex-specific genes. *SRY*, the gene responsible for sex determination, was not detected in any embryo. On the other hand, other genes associated to the Y chromosome, such as *EIF1AY* and *DDX3Y*, were expressed in all embryos except blastocyst 3. Furthermore, *XIST*, a gene involved in X-chromosome inactivation in female cells, was expressed at high levels only in blastocyst 3. This suggests that blastocyst 3 is a female embryo while the others are male. The high male to female ratio could be explained by the tendency of male embryos to grow faster under in vitro culture conditions and it is possible that selecting for more advanced embryos at the time of collection resulted in a male bias.

We also noticed that the clustering of samples based on all genes analyzed discriminated blastocyst 3 from the other embryos, suggesting that global levels of gene expression can discriminate embryo gender. In support of this, one third of expressed genes were previously found to be differentially regulated when comparing male and female embryos using microarray analysis of bovine blastocysts produced with sexed semen [38].

We thus performed a statistical analysis comparing blastocyst 3 to the rest. A total of 168 genes were differentially expressed between the males and female blastocyst at an adjusted P-value of < 0.05 and 2 fold change. Of these, 144 were overexpressed in the female embryo. Among them, 47 genes (33%) were located to the X chromosome, a proportion much higher than expected by chance since the X chromosome only contains 4.6% of annotated genes. Moreover, of the overexpressed genes in males, 5 of the 24 genes (21%) were homologous to Y-linked genes (*ZRSR2Y*, *DDX3Y*, *OFD1Y*, *EIF2S3Y*, and *UTY*). This is consistent with results from a microarray study comparing male and female bovine embryos where differentially expressed genes were enriched to the sex chromosomes [38]. Interestingly, the microarray study found most differences to be lower than 2 fold-changes, whereas our study indicated fold-changes ranging from 2.25 to > 589. These discrepancies are probably associated with the larger dynamic range of RNA-seq versus microarrays and suggest that further analysis using RNA-seq in female and male embryos would be informative. We also compared our results to the list of differentially expressed genes reported by Bermejo-Alvarez et al. [38] (Additional file 2: Figure S2). For genes reported overexpressed in female embryos, we found that among the 66 genes with a common identity to ours, 54 (82%) had a fold change > 1.5 in our data. Similarly, all matching genes reported upregulated in males by Bermejo-Alvarez et al. were also higher in our male samples compared to the female one. Finally, among transcripts validated by qPCR in the aforementioned study, all the matching genes (n = 13) differed in the same direction in our data, with 10 of them having a P-value < 0.05. The similarities in the results of these two studies reinforce the notion that global gene expression analysis can differentiate between embryos of different sexes.

No functional categories were enriched in the group of genes overexpressed in male embryos. Among female overexpressed genes, no GO molecular process was significantly overexpressed while cell adhesion (*HAPLN4*, *PGM5*, *PCDHB6*, *CLSTN3*, *CTGF*, *ICAM5*, and *IZUMO1*), glucose metabolism (*PPP1R3C*, *LDHA*, *PGM5*, and *PGK1*) and cell motility (*CTGF*, *ARID5B*, *ATP1A3*, *TNP1*, and *PRKG1*) were overrepresented categories among biological functions (P < 0.05).

### SNP identification in single embryo transcriptomes

Next generation transcriptome sequencing allows for identification and discovery of genetic variants located in transcribed regions of the genome. Genetic variation in gene coding sequences has a higher potential to affect phenotypic characteristics. To investigate the usefulness of single embryo transcriptome data for detecting known and novel genetic variation we used CLC Genomic Workbench SNP identification tool. Reads were mapped to the UMD3.1 reference genome, and nucleotides within reads that differed from the reference were identified as SNP. An SNP was considered homozygous in the sample if only a variant allele was present. SNP were considered heterozygous if both a variant and the reference nucleotide were detected at a given position. SNP validation was performed in-silico by matching putative SNP positions to known bovine dbSNP entries (Ensembl Bovine GVF release 67). SNP were considered validated when a corresponding dbSNP entry was found and the variant nucleotide identities coincided exactly with our data. The random chance of exact variant matching is only 12.5% for heterozygous SNP and 33% for homozygous SNP. Initially, variants were called if the SNP was covered by more than 10 reads and, in the case of heterozygous SNP, if the lower expressed allele was present in at least 4 reads. Roughly, half of the SNP that were identified matched a dbSNP variant position, and out of these more than 99% shared the same nucleotide alleles between our samples and the reference database. When performing the validation it was noticed that a higher proportion of homozygous SNPs corresponded to already known SNP compared to heterozygous SNP. However, when an SNP was found in both our data and dbSNP, the allelic variants coincided in > 99% of the cases for both homozygous and heterozygous SNP. We interpreted these findings as a suggestion that the rate of false SNP discovery is higher in heterozygous SNP (fewer proportion of detected SNP is already known). This could be attributed to a lower threshold required to call the lower variant in a heterozygous SNP, since only 4 reads are required for the lower allele to be identified as a SNP versus 10 reads for homozygous SNP. We therefore examined how SNP coverage related to validation rate in order to estimate the minimum sequencing depth to accurately call new SNP. The proportion of SNP matching dbSNP across a spectrum of coverage levels was calculated and found to increase appreciably from 4 to 14 reads and then plateau afterwards through 30 reads per variant (Additional file 3: Figure S3). Therefore, 14 reads was selected as the minimum coverage level for novel SNP (not found in current database). Increasing the threshold for SNP discovery resulted in fewer sequencing errors being determined as SNP. It is still possible however that certain genes with high expression levels could accumulate a sufficient number of errors to reach the minimum threshold for SNP identification.

To investigate this possibility, SNP detected in mitochondrial genes were examined, because these genes were highly expressed and because the mitochondrial genome is assumed to be homozygous [39]. We assumed that the presence of heterozygosity in the mitochondrial genome is the result of sequencing errors and therefore represents false SNP discovery. When heterozygous SNP in mitochondrial genes were evaluated, a total of 392 unique SNP with an average of 109 per sample were observed. The frequency of the minor allele averaged 2.3 ± 0.2%, and was consistent for each of the embryos. More than 95% of the SNP in mitochondrial genes consisted of SNP with the minor allele representing less than 15% of the reads. Based on this, 15% was chosen as the lower limit cutoff for minor alleles to limit the probability (< 5%) that sequencing errors in highly expressed genes influence heterozygous SNP discovery and allele specific expression analysis. Thus, coverage thresholds used should be carefully considered in order to minimize calling of sequencing artifacts in the interest of maximizing variation coverage. It should also be noted that the potential for heteroplasmy in mtDNA [40,41] makes the lower threshold for SNP detection very conservative.

**Figure 5:**
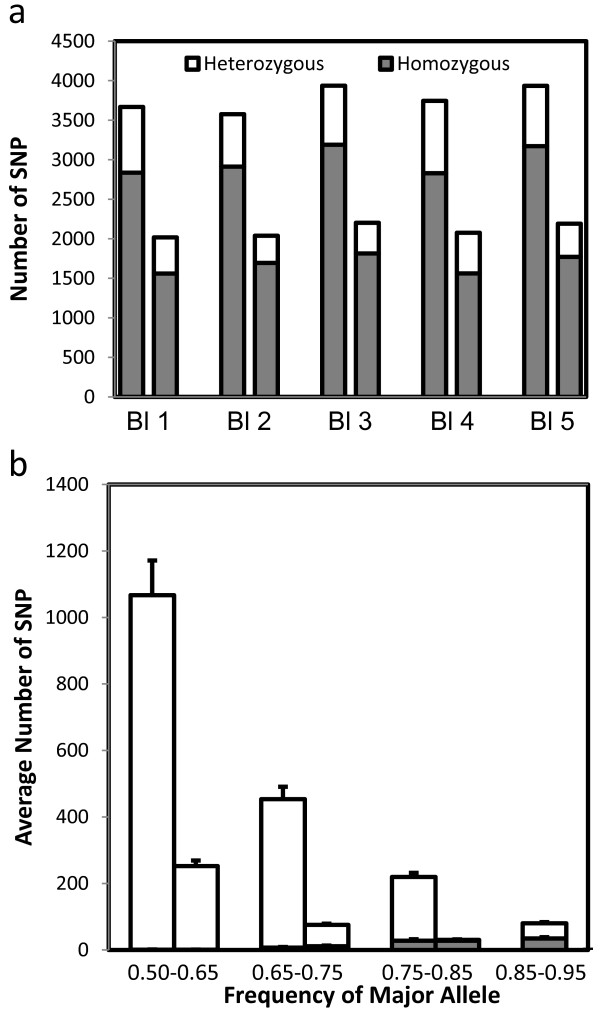
**Allelic expression analysis in single bovine embryos. a**) Number of SNP detected in each sample. Left bar indicates the total number of SNP detected. Right bar, represents the number of SNP that matched dbSNP variants by position. More than 99% of those matches agreed on nucleotide identity. **b)** Allelic bias in heterozygous SNP expression**.** Number of SNP expressed at different major allelic frequencies. Average number of validated (left bar) and putative (right bar) SNPs are presented separate. The number of SNP with statistically significant imbalance (FDR < 0.01 by X^2^ test) is indicated within their respective group (shaded area). The 85-95% frequency only includes validated SNPs.

The optimized parameters were only used for identification of de-novo SNPs, while SNP detected with the less stringent set of parameters that matched dbSNPs were considered valid. Using these criteria, a total of 10,734 unique SNP were identified, 2,525 (23.50%), of which were heterozygous in at least one sample (Figure 5). A large proportion of detected SNP (55.7%) were present in the dbSNP database. This suggests that SNP identified from the transcriptome sequencing of single embryos are highly reliable and can be used to discover novel SNP at genome coding sequences and for analysis of allele specific expression.

### Allele specific expression analysis

Analysis of allele specific expression requires the presence of heterozygous SNP in the transcribed portions of a gene. Among the 2,524 heterozygous unique SNP that were identified, 1,018 known genes were represented. Allelic imbalance (AI) was determined for these SNP when the proportion of reads assigned to one allele was higher than 65%. We found that forty percent of expressed heterozygous SNP demonstrated an allelic imbalance (Figure 5a). A similar proportion was observed for novel SNP, although in this category the highest allowed ratio was 85%. The statistical significance of these imbalances was assessed in order to diminish the presence of false positives. Statistical significance of expressed AI bias was determined if the read distribution was significantly different than a theoretical 50:50 distribution, as determined by a Chi^2^ test adjusted for FDR (adjusted p < 0.01). Statistical thresholds reduced the number of SNP with AI to 473 (19% of all heterozygous SNP detected) within 176 unique genes (Figure 5b). The stringency of the criteria used to assess these significant biases, while minimizing false positives, restricts the ability to create a comprehensive list of SNP given that only those with high coverage provided sufficient information for statistical analysis. Indeed, 82% of genes with significant AI were among the top 20th percentile of expression levels, demonstrating the necessity of sufficient read depth for ASE detection. Increased sequencing depth would likely allow discrimination of AI in more genes and also provide more statistical power to determine the significance of low level AI.

In order to estimate the number of genes in blastocysts that could possibly express AI, we determined the proportion of expressed AI with statistical significance within a subset of high coverage genes. Of 478 genes containing SNP with high coverage (>62 reads per SNP), 160 were found to also have SNP expressing significant AI (317). We estimate that with sufficient sequencing depth, ~33% of expressed genes in bovine blastocyst genes could have AI with a major allele frequency > 65%. Differences in epigenetic reprogramming of maternal and paternal genomes during preimplantation development could result in allelic skewing of embryonic gene expression. AI dependent on parental origin and cellular lineage may also be important for embryonic development [42,43]. However, it is not known at this time if the preferentially expressed alleles have a common parental origin. GO enrichment analysis of genes containing SNP with statistically significant AI identified major cellular constitutive elements as overrepresented categories. These included GO terms such as cytoskeleton, basolateral membrane, focal adhesion, non-membrane-bounded organelles, organelle lumen and macromolecular complex organization, among others. The enrichment of genes to these specific cellular elements is not surprising given that they are highly expressed cellular components and, as stated above, detection of imbalance is greatly aided by high coverage or expression.

Imprinting is one phenomenon that leads to allelic expression imbalance, with typically only one specific parental allele being expressed. The approach used to detect imbalances is based on the expressed genes without knowledge of the individual embryo genotype, and therefore in cases of monoallelic expression it would be expected that no heterozygous alleles would be identified. Among 37 genes known to be imprinted in cattle, human and/or mouse, 17 were expressed in bovine blastocysts at RPKM > 0.3 [44,45]. Expressed heterozygous SNP were found in 9 of these genes (17 SNP in total; Table 6). Lack of heterozygous SNP in other imprinted genes could indicate mono-allelic expression. Allelic biases (>65% reads corresponding to one allele) were observed in 16 out of the 17 detected heterozygous SNP (Table 6). Because of low imprinted gene expression and low coverage of the SNP, only 6 candidate imprinted SNP from 4 genes (*ASB4*, *BLCAP*, *NNAT*, and *NAT15*) had AI with statistical significance (Chi^2^ adjusted p < 0.01). Identification of imbalanced alleles in most of the known imprinted genes within our dataset further corroborates the utility of RNA-seq in characterization of AI.

**Table 6:** List of known imprinted genes with expressed SNP

**Gene name**	**Species in which imprinted**	**Bovine gene Ensembl ID**	**# Heterozygous SNP identified**	**Frequency of major allelic variant (%)**
*NNAT*	Bovine	ENSBTAG00000003212	3	82, 89*, 86*
*NAP1L5*	Bovine	ENSBTAG00000010128	1	59
*NNAT*	Bovine	ENSBTAG00000045928	1	82
*UBE3A*	Murine	ENSBTAG00000002487	1	69
*NAT15*	Murine	ENSBTAG00000004875	1	91*
*GAB1*	Human	ENSBTAG00000002813	5	64, 71, 75, 78, 67
*BLCAP*	Human	ENSBTAG00000003209	2	89*, 86*
*COPG2*	Human	ENSBTAG00000017245	2	67, 68
*ASB4*	Human	ENSBTAG00000018185	1	84*

* Chi-squared p < 0.01 difference from 50:50 expression of both alleles.

## Conclusions

This study reports the analysis of individual bovine embryo transcriptome sequencing, providing details for amplification procedures of low RNA input samples and an analysis pipeline that examines differences in gene expression profiles, identifies novel SNP and determines instances of allelic imbalance. RNA-seq analysis in single embryos allows for discrimination of embryo gender and provided the opportunity to characterize individual variability in gene expression. SNP analysis of individual samples demonstrates the use of RNA-seq to identify embryo-specific variation for association studies and ASE that could represent novel layers of developmental regulation subject to influence by AI. Our data suggests that AI is prevalent in bovine blastocysts. RNA-seq analysis of the individual embryonic transcriptome is feasible and presents valuable insights into gene expression, variation and regulation of the early developmental transcriptome.

## Methods

### Sample preparation and RNA extraction

Bovine oocytes from Holstein animals were obtained from commercial suppliers and matured overnight in a portable incubator. Matured oocytes were used for in vitro fertilization (IVF) with semen from a Holstein bull, as previously reported [46]. IVF embryos were cultured for a period of 7 days in KSOMaa medium supplemented with BSA (4 mg/mL), and 5% fetal bovine serum added after 3 days in culture. Blastocyst stage embryos were collected, treated for 2 minutes with pronase (1 mg/mL) to remove the zona pellucida (ZP) and any contaminating cumulus cells and individually stored in 20 μL of Extraction Buffer from the PicoPure RNA Isolation kit (Applied Biosystems, Carlsbad, CA) at −80°C. RNA was extracted using the PicoPure RNA Isolation Kit, including DNAse treatment, following manufacturer’s instructions but with a modified RNA elution step. In order to achieve a high concentration of RNA, elution was performed using 7 μL of DEPC-treated water and the eluate was run through the column one more time after the initial elution. RNA quantification and quality control were performed using a High-Sensitivity RNA Chip with the Experion microfluidic gel system (Bio-Rad, Hercules, CA). RNA quality was evaluated by examining the 18 and 28S ribosomal subunits, quantity, and RNA quality indicator (RQI) score.

### RNA amplification and sequencing library preparation

Total RNA was used as input for the Ovation RNA-seq V1 kit (NuGen, San Carlos, CA). cDNA output was analyzed for correct size distribution with an Experion Standard Sensitivity RNA chip and quantified using a Qubit Fluorometer (Invitrogen, Carlsbad, CA). Amplified cDNA with a size distribution of ~200-500 bp was considered acceptable. Then, 200 ng of cDNA from each sample were used with the NuGen Encore NGS Library I kit to produce sequencing libraries sized between 200–400 bp and lacking primer dimer peaks. Libraries were sequenced at the UC Davis Genome Center Sequencing Core with an Illumina Genome Analyzer IIx as 40 bp single reads (software version RTA 1.8). Quality control of reads was performed using the Fastqc module from Galaxy (http://main.g2.bx.psu.edu/) [47-49].

### Read alignment and gene expression analysis

Reads were mapped to bovine genome assemblies using CLC Genomics Workbench 4.7 software (CLC bio, Aarhus, Denmark). To quantify gene expression, the RNA-seq Analysis tool was used as previously described [14] allowing for no more than 2 mismatches per read. Annotations were downloaded from NCBI or ENSEMBL for bovine genome builds Btau4.0, Btau4.2 and UMD3.1. The non-coding RNA database RFAM 10.1 [50] was used for diagnostic alignments of rRNA species.

### Functional annotation of transcripts

Expressed transcripts were sorted by average RPKM (reads per kilobase of exon model per million mapped reads) and divided into equally-proportioned groups. Genes with RPKM lower than 0.4 were not included in this analysis, leaving a total of 9,490 genes for functional annotation and divided into 5 groups. Ensembl Gene IDs from each group were uploaded to the DAVID Functional Annotation Tool (http://david.abcc.ncifcrf.gov/; Version 6.7) and analyzed for enrichment using Functional Annotation Clustering [51,52]. The classification stringency was set to Medium and other settings were default parameters. Cluster analysis outputs from the 5 groups were ranked by Enrichment Score and the top 10 clusters summarized based on general cellular components related to their respective functions.

### SNP and ASE analysis

SNP were identified using the SNP Detection tool from CLC Genomics Workbench on reads aligned to the UMD3.1 bovine genome assembly. Stringency parameters for the analysis were set as previously described [14]. An SNP was considered homozygous in the sample if a nucleotide was different from the one in the same position in the reference genome (UMD3.1). A heterozygous SNP was that in which the two alleles were present in the sample. Identified SNP were compared with those in the ENSEMBL bovine SNP database based on genomic position and allelic variants. SNP not present in the database were considered novel. For allele specific expression (ASE) analysis the proportion and ratio of uniquely mapped reads, excluding duplicated reads, containing each allelic variant in a heterozygous SNP were calculated. Allelic imbalance (AI) was defined as a statistically significant deviation from the expected 50:50 ratio (Chi-square P < 0.01) and a frequency of the most abundant allele greater than 65%.

### Availability of supporting data

The data sets supporting the results of this article are available in the GEO repository, under accession GSE44023.

## Competing interests

The authors declare that they have no competing interests.

## Authors’ contributions

JLC, GGK and PJR performed the experiments; JLC, GGR, PJR analyzed the data; JFM and PJR planned the experiment; JLC and PJR prepared the manuscript. All authors read and approved the final manuscript.

## Supplementary Material

Additional file 1: Figure S1 Principal component analysis (PCA) of RPKM levels in single IVF blastocysts, pool of blastocysts from Huang and Khatib (2010) data, and milk somatic cells. Single and pool blastocyst datasets clustered close together while the somatic cells were further apart. This result supports the validity of single embryo RNA-seq analysis. Click here for file

Additional file 2: Figure S2 Comparison of male vs. female embryo differential gene expression between RNA-seq and microarray results reported by Bermejo-Alvarez et al. 2010. Click here for file

Additional file 3: Figure S3 The proportion of SNP validated by dbSNP as a proportion of the total detected was compared across coverage levels ranging from 4 to 30. Proportion validated increased moderately (approximately 7%) from 4 to 10, but this trend reached saturation at coverage of 14 (>55% validation). Minimum coverage for analysis was based on this saturation threshold. Click here for file
