# Supplementary figures and images for: RNA-seq analysis of single bovine blastocysts
Source: BMC Genomics. 2013 May 25;14:350. doi: 10.1186/1471-2164-14-350 (PMC3668197; doi:10.1186/1471-2164-14-350)

Projection scatter plot

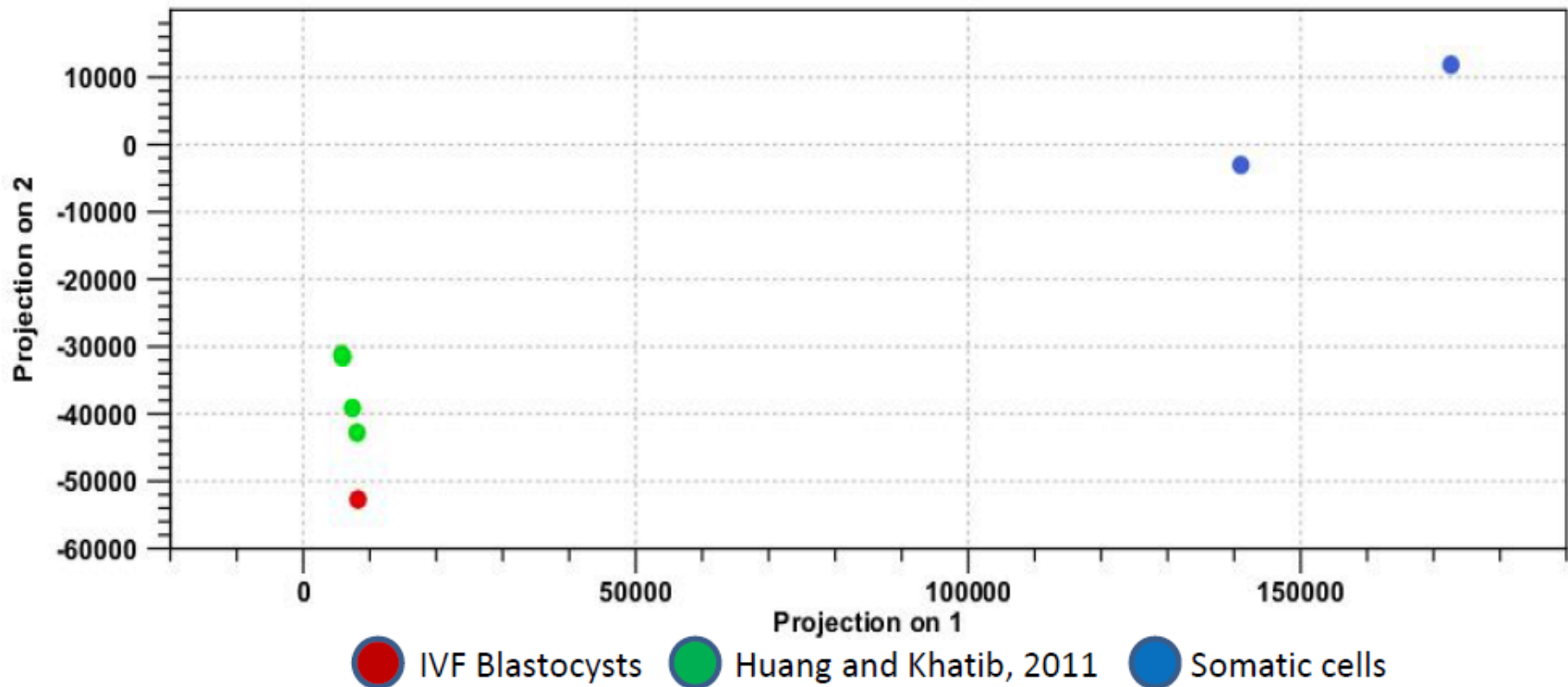

Supplement: Additional file 1: Figure S1 — Principal component analysis (PCA) of RPKM levels in single IVF blastocysts, pool of blastocysts from Huang and Khatib (2010) data, and milk somatic cells. Single and pool blastocyst datasets clustered close together while the somatic cells were further apart. This result supports the validity of single embryo RNA-seq analysis. [file 1471-2164-14-350-S1.pdf]

# Proportion of Validated SNP by Coverage

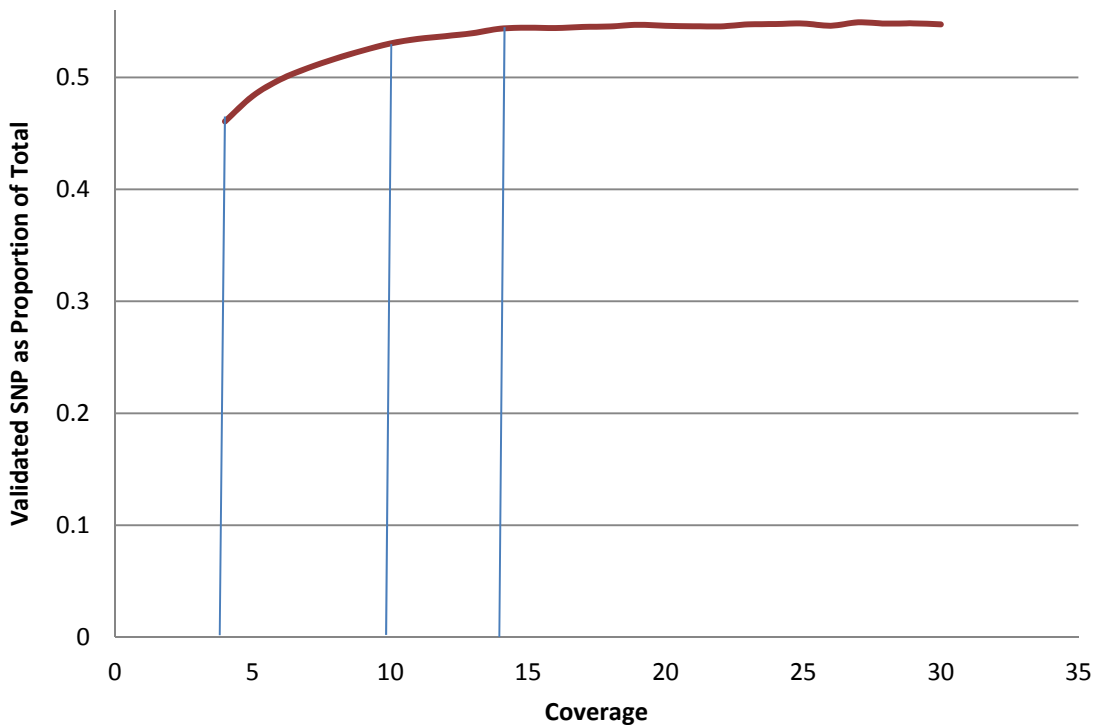

Supplement: Additional file 3: Figure S3 — The proportion of SNP validated by dbSNP as a proportion of the total detected was compared across coverage levels ranging from 4 to 30. Proportion validated increased moderately (approximately 7%) from 4 to 10, but this trend reached saturation at coverage of 14 (>55% validation). Minimum coverage for analysis was based on this saturation threshold. [file 1471-2164-14-350-S3.pdf]
