# Supplementary material for: RNA-seq analysis of single bovine blastocysts
Source: BMC Genomics. 2013 May 25;14:350. doi: 10.1186/1471-2164-14-350 (PMC3668197; doi:10.1186/1471-2164-14-350)
Supplement: Additional file 2: Figure S2 — Comparison of male vs. female embryo differential gene expression between RNA-seq and microarray results reported by Bermejo-Alvarez et al. 2010. [file 1471-2164-14-350-S2.pdf]

Genes upregulated in Female embryos according to Bermejo-Alvarez et al. Supplementary Table 2

Gene Symol	Fold-change	RNA-Seq	
		Fold-change	P-value
CAPN6	4.07	7.30	0.000698
GSTM3	3.94	8.46	0.005842
FMR1NB	3.10	4.99	1.34E-06
BEX2	2.81	1.78	0.237164
NGFRAP1	2.80	2.56	0.092733
LOC618696	2.77	2.25	0.008966
SAT1	2.73	5.84	1.39E-06
HSPA1A	2.68	3.14	0.001354
RNASE1	2.47	8.52	0.000111
PAGE4	2.45	1.50	0.123876
SRPX2	2.43	13.01	3.67E-06
RESP18	2.39	1.63	0.245028
LDOC1	2.36	3.36	0.010835
HSPA1A	2.33	3.14	0.001354
MATN4	2.28	80.22	3.17E-06
PHLDA2	2.28	66.71	0.000813
DHRS9	2.27	5.51	0.001709
UCHL1	2.27	9.22	0.001175
UBD	2.23	-2.50	0.662727
HSPA1A	2.21	3.14	0.001354
BDH2	2.20	3.76	0.03585
LOC527068	2.18	0.00	1
MAOB	2.18	2.64	0.056082
MAGEH1	2.16	3.78	0.027809
LGALS3BP	2.15	2.18	0.134484
CLU	2.12	1.76	0.147409
APOL3	2.11	-1.66	0.949156
PRPS1	2.09	1.65	0.164047
BRWD3	2.08	2.36	0.001292
TUBA4A	2.06	4.65	0.02005
TRAPPC2	2.02	4.58	0.006648
TUBA4A	2.00	4.65	0.02005
UPK1A	1.99	3.42	0.056039
UBQLN2	1.97	2.19	0.146924
ZAP70	1.96	1.33	0.278688
SULT1A1	1.96	-2.95	0.88327
THEM4	1.95	1.67	0.141262
PDK3	1.95	2.04	0.043761
TNNI3	1.95	1.25	0.451533
RNF113A	1.92	1.74	0.13723
SNX12	1.92	1.65	0.084861
TCEAL8	1.91	2.00	0.011906
TM4SF1	1.91	6.80	5.33E-05
SLITRK2	1.89	3.36	0.019761
MID1IP1	1.87	-1.06	0.669026
STEAP2	1.87	-1.69	0.6686
CITED1	1.86	2.00	0.073831
LY6G6C	1.85	1.53	0.281787
HSPB8	1.84	2.91	0.08853
ABHD4	1.84	6.22	0.001209
ATRX	1.83	1.99	0.001776
CXCL16	1.82	5.56	0.016184
MCTS1	1.81	1.67	0.249703
S100A4	1.81	5.72	0.021142
ALDOC	1.81	1.13	0.401329
FAM122B	1.80	1.84	0.121164
TSPAN6	1.79	6.99	1.58E-14
CETN2	1.79	2.05	0.023918
UBE2A	1.78	2.67	0.013911
MAGED2	1.78	1.61	0.305265
WDR44	1.77	2.81	1.6E-05
MAP3K12	1.77	-1.88	0.424023
APEX2	1.77	-2.43	0.135417
S100A11	1.76	2.43	0.040479
NKRF	1.75	2.95	0.053981
FUNDC1	1.75	1.99	0.075586

Genes downregulated in Female embryos according to Bermejo-Alvarez et al. Supplementary Table 3

Gene Symol	Fold-change	RNA-Seq	
		Fold-change	P-value
LAMA1	−1.94	-2.00	0.03623
BPHL	−1.76	-1.29	0.875077
P2RX4	−1.70	-2.00	0.194187
SLC6A20	−1.67	-1.96	0.027027
SHPK	−1.55	-3.53	0.152872
ABCB6	−1.44	-1.42	0.601065
HSD17B8	−1.44	-1.51	0.975492
PRMT7	−1.43	-1.32	0.53449
SNAPC5	−1.43	-1.28	0.936773
HN1L	−1.43	-1.48	0.351526
LOC516579	−1.42	-1.22	0.743871
COQ6	−1.42	-1.17	0.922727
FBXL6	−1.42	-5.51	0.842894
LOC514296	−1.41	-3.33	0.150904

Genes analyzed by Q-PCR in Bermejo-Alvarez et al.

	Fold-change		RNA-Seq	
	qPCR	Array	Fold-Change	P-value
FMR1NB	3.02	3.1	4.99	1.34292E-06
CAPN6	4.13	4.07	7.30	0.000698058
GSTM3	3.52	3.94	8.46	0.005842053
SAT1	2.83	2.73	5.84	1.38741E-06
BEX2	2.32	2.8	1.78	0.237164339
SRPX2	1.75	2.43	13.01	3.66713E-06
PGRMC1	1.52	1.62	1.44	0.280452497
UBE2A	2.56	1.78	2.67	0.01391063
LAMA1	−1.76	−1.93	-2.00	0.036229903
DNMT3A	−1.75	−1.19	-1.64	0.051687763
YZRSR2	−inf	−12.01	−inf	1.43836E-16
DDX3Y	−inf		-407.79	5.70185E-25
EIF2S3Y	−inf		-48.56	3.69497E-20

Reference:
Bermejo-Alvarez P, Rizos D, Rath D, Lonergan P, Gutierrez-Adan A: Sex determines the expression level of one third of the actively expressed genes in bovine blastocysts. Proc Natl Acad Sci USA 2010, 107(8):3394–3399.
